# Investigating the regulation of the miR-199a-3p/TGF-β/Smad signaling pathway by BSHXF drug-containing serum combined with ADSCs for delaying intervertebral disc degeneration

**DOI:** 10.3389/fphar.2025.1583635

**Published:** 2025-04-28

**Authors:** Enxu Liu, Yu Sun, Lei Yang, Haobo Jiang, Fei Sun, Long Chen, Jiahao Duan, Shaofeng Yang

**Affiliations:** ^1^ Hunan University of Traditional Chinese Medicine, Graduate School, Changsha, Hunan, China; ^2^ The First Affiliated Hospital of Hunan University of Traditional Chinese Medicine, Department of Orthopaedics, Changsha, Hunan, China

**Keywords:** intervertebral disc degeneration, miR-199a-3p/TGF-β/Smad signaling pathway, adipose-derived stem cells, oxidative stress, extracellular matrix synthesis

## Abstract

**Background:**

Intervertebral disc degeneration (IDD) significantly contributes to low back pain (LBP), yet effective treatment options are scarce. BSHXF, a classical traditional Chinese medicine formula, demonstrates dual pharmacological actions: tonifying kidneys, strengthening bones, activating blood circulation, and resolving stasis. It has been widely used in IDD management. Given its potential, combining BSHXF with miRNA regulation and stem cell therapy may enhance therapeutic outcomes by targeting molecular and cellular pathways underlying IDD pathogenesis.

**Aim of the study:**

IDD is recognized as one of the primary causes of low back pain, yet effective therapeutic interventions for this condition remain limited. This study explores the role of BSHXF drug-containing serum combined with adipose-derived stem cells (ADSCs) in slowing IDD progression via the miR-199a-3p/TGF-β/Smad signaling pathway. By comprehensively investigating the synergistic effects of this combination therapy, we aim to propose a novel multi-target strategy that addresses the complex pathogenesis of IDD.

**Materials and Methods:**

This study employed a combination of *in vivo* and *in vitro* models. An IDD model was induced in rat caudal intervertebral discs through needle puncture, while an oxidative stress-induced ADSCs injury model was created *in vitro* using tert-butyl hydroperoxide (T-BHP). Cell viability was measured with the CCK-8 assay. Cell cycle distribution and mitochondrial reactive oxygen species (ROS) levels were assessed using flow cytometry. Cellular senescence was assessed using SA-β-galactosidase staining. Lactate dehydrogenase (LDH) activity was quantified to evaluate cellular damage. Differentiation into nucleus pulposus-like cells was assessed using immunofluorescence double staining for CD73 and COL2A1. ELISA was used to measure inflammatory cytokines (TNF-α, IL-1β, IL-4, IL-10) in cell supernatants. miR-199a-3p expression was determined using RT-qPCR. Western blotting was employed to quantify COL2A1, SOX9, and ACAN protein levels, reflecting nucleus pulposus-like differentiation and extracellular matrix (ECM) synthesis capacity. Western blotting was employed to assess pathway activity by analyzing the protein expressions of TGF-β1, Smad2, Smad3, and their phosphorylated forms, P-Smad2 and P-Smad3. *In vivo* experiments assessed histopathological degeneration through hematoxylin-eosin (HE) and Safranin O-Fast Green staining. Immunohistochemistry (IHC) analyzed COL1A1 and COL2A1 expression levels. RT-qPCR quantified miR-199a-3p expression. Western blotting was employed to assess the expression levels of TGF-β1, Smad2, Smad3, P-Smad2, and P-Smad3 for pathway regulation evaluation.

**Results:**

Our experimental results demonstrated that serum containing BSHXF significantly alleviated T-BHP-induced oxidative stress, improved the cellular microenvironment, promoted ADSCs proliferation, and decelerated cellular senescence. Further mechanistic analysis revealed that BSHXF significantly activated the TGF-β/Smad signaling pathway, driving the differentiation of ADSCs into nucleus pulposus-like cells and restoring normal cell cycle progression. Overexpression of miR-199a-3p inhibited the TGF-β/Smad pathway, leading to ECM degradation and elevated expression of inflammatory factors (TNF-α, IL-1β). In contrast, BSHXF restored TGF-β/Smad pathway activity by downregulating miR-199a-3p expression. *In vivo* experiments demonstrated that miR-199a-3p overexpression exacerbated IDD, characterized by reduced COL2A1 expression, elevated COL1A1 levels, and increased disc fibrosis. BSHXF intervention markedly attenuated IDD progression by downregulating miR-199a-3p expression, reducing disc fibrosis, and effectively restoring collagen expression.

**Conclusion:**

BSHXF activated the TGF-β/Smad pathway to promote the differentiation of ADSCs into nucleus pulposus-like cells. It exerted protective effects by alleviating oxidative stress damage, improving the microenvironment, delaying senescence, and enhancing cellular functions. This study is the first to reveal that miR-199a-3p overexpression exacerbates intervertebral disc fibrosis and degeneration. BSHXF restored TGF-β/Smad pathway activity by downregulating miR-199a-3p expression, thereby improving disc structure and function. This integrated approach offers a novel multi-target intervention strategy for IDD, demonstrating significant therapeutic potential.

## 1 Introduction

IDD is widely acknowledged as the predominant etiology of LBP, accounting for approximately 40% of cases. This condition imposes a substantial global health and socioeconomic burden (Clark and Horton, 2018; Wang et al., 2022a). Therefore, understanding its etiology and pathogenesis is critical for developing and maintaining effective therapeutic strategies (Parenteau et al., 2021). IDD is associated with multiple pathological changes, most notably ECM degradation, inflammatory responses, and cellular apoptosis (Clouet et al., 2009). However, no effective and reliable therapies currently exist, primarily due to the incomplete understanding of its pathogenic mechanisms (Cazzanelli and Wuertz-Kozak, 2020).

BSHXF comes from the altered traditional formula Qing’e Wan, recorded in the Song Dynasty’s “Taiping Huimin and Agent Bureau” (Lu et al., 2018). This herbal formulation contains key components such as Eucommia ulmoides Oliv (Du Zhong), Cullen corylifolium (Bu Gu Zhi), Achyranthes bidentata Blume (Huai Niu Xi), Salvia miltiorrhiza Bunge (Dan Shen), Chaenomeles speciosa Nakai (Mu Gua), and Clematis chinensis Osbeck (Wei Ling Xian). The dosage of BSHXF herbs is provided in Table 1. Traditional Chinese medicine identifies deficiency in kidney function and blood stagnation as the main causes of IDD, with therapies focusing on tonifying the kidneys and activating blood as essential treatment principles. BSHXF is formulated to have dual effects: kidney-tonifying, bone-strengthening, blood-activating, and stasis-resolving properties (Zhu et al., 2020). Clinical observations showed that oral administration of BSHXF decoction significantly alleviated symptoms of lumbar intervertebral disc degenerative pain, with sustained therapeutic efficacy throughout the follow-up period (Zhu et al., 2017). Our previous research demonstrated that BSHXF ameliorates the degenerative microenvironment in IDD, boosts nucleus pulposus cells (NPCs) proliferation, and decelerates IDD progression (Yang et al., 2019). Furthermore, UPLC-Q-TOF-MS was employed to detect bioactive components in serum that contains BSHXF. A total of 70 hematogenous constituents and their breakdown products were detected, comprising 38 prototype compounds. BSHXF drug-containing serum was found to regulate the signaling pathway involving TGF-β/Smad, thereby enhancing the differentiation of ADSCs into NPCs. It also reduced oxidative stress-induced cell cycle arrest in NPCs, enhanced NPCs proliferation, delayed NPC senescence, and improved the surrounding pathological microenvironment (Duan et al., 2023). Furthermore, we assessed changes in the expression levels of miR-99a, miR-99b, miR-146a-5p, miR-146b-5p, and miR-199a-3p in ADSCs treated with BSHXF-containing serum using RT-qPCR. The results demonstrated that BSHXF-containing serum downregulated miR-199a-3p expression in ADSCs, with miR-199a-3p exhibiting the most significant reduction among the tested miRNAs. These findings suggest that miR-199a-3p may play a pivotal role in regulating the TGF-β/Smad pathway and modulating cellular proliferation and differentiation (Supplementary Figure S1).

**TABLE 1:** Herbal formula of BSHXF.

Chinese name	English name	Material	Scientific name of the herb	Place of origin	Quantities(g)^*^
Du Zhong	Eucommia Cortex	Bark	Eucommia ulmoides Oliv	Hunan, China	15
Bu Gu Zhi	Psoraleae Fructus	Fruit	Cullen corylifolium (L.)	Sichuan, China	10
Huai Niu Xi	Achyranthes bidentata	Root	Achyranthes bidentata Blume	Henan, China	10
Dan Shen	Salviae Miltiorrhizae Radix et Rhizoma	Root and rhizome	Salvia miltiorrhiza Bunge	Shandong, China	12
Mu Gua	Chaenomelis Fructus	Fruit	Chaenomeles speciosa (Sweet) Nakai	Hunan, China	9
Wei Lin Xian	Clematidis Radix Et Rhizoma	Root and rhizome	Clematis chinensis Osbeck	Liaoning, China	10

The plant name have been checked with http://www.theplantlist.org.

* Dry weight of the restorative material.

Numerous therapeutic approaches are currently under investigation, with cell-based therapies and miRNA-targeted treatments emerging as the most promising options (Clouet et al., 2019; Henry et al., 2018). Stem cells, characterized by their pluripotency and self-renewal capacity, participate in fundamental biological processes including cellular differentiation, proliferation, angiogenesis, oxidative stress responses, inflammatory regulation, and ECM synthesis (Costa et al., 2021). Stem cell therapies have been extensively investigated for treating degenerative musculoskeletal disorders (Elashry et al., 2022). In recent years, stem cells derived from the nucleus pulposus (NP), bone marrow, and adipose tissue have demonstrated significant clinical potential for IDD by modulating key signaling pathways involved in its pathogenesis (Du X et al., 2023). ADSCs are considered optimal progenitor cell sources for tissue engineering applications, owing to their high tissue accessibility, simplified isolation protocols, and inherent low immunogenic potential (Zhang et al., 2022). As important regulatory molecules, miRNAs are involved in key cellular functions such as proliferation, differentiation, and apoptosis (Guil and Esteller, 2009; He and Hannon, 2004). Dysregulated miRNA expression has been implicated in various human musculoskeletal disorders (Ali et al., 2021; Jiang et al., 2023). Recent studies have increasingly identified miRNAs as pivotal regulators of IDD progression, influencing cellular proliferation, apoptosis, ECM degradation, senescence, and inflammation (Wang X. et al., 2020; Wang et al., 2022b; Xiang et al., 2022). Although prior research highlights the critical role of the TGF-β/Smad pathway in disc homeostasis, the mechanisms by which miR-199a-3p regulates this pathway and how herbal components improve the stem cell microenvironment remain unclear. This study addresses two core scientific questions: (1) Does miR-199a-3p exacerbate IDD by suppressing the TGF-β/Smad pathway? (2) Can BSHXF-containing serum restore pathway activity and enhance ADSCs therapeutic efficacy through miR-199a-3p regulation? Accordingly, this study proposes a synergistic strategy integrating ADSCs’ regenerative potential, miR-199a-3p targeting, and BSHXF-containing serum. Specifically, BSHXF downregulates miR-199a-3p expression, activates the TGF-β/Smad pathway, enhances ADSCs survival, differentiation, and ECM synthesis, and remodels the degenerative microenvironment, thereby overcoming the therapeutic limitations posed by pathological microenvironment-stem cell interactions.

## 2 Materials and Methods

### 2.1 Reagents

DMEM low-glucose medium was obtained from Procell (China), and T-BHP was obtained from Sigma. (United States). PBS, CCK-8 assay kit, trypsin digestion solution, fetal bovine serum (FBS), penicillin-streptomycin mixed solution, multiplex immunofluorescence double labeling reagent, DAPI, agarose, DNA gel loading buffer (6×), Tris-acetate electrophoresis buffer (50×TAE), DEPC-treated water (0.1%), cell freezing medium, neutral balsam, hematoxylin, eosin, Safranin O-Fast Green staining kit, DAB kit, urea-trypsin antigen retrieval solution, COL2A1, SOX9, ACAN, Smad2, Smad3, P-Smad2, and P-Smad3 were obtained from Abiowell (China). The ROS assay and β-galactosidase staining kits were sourced from Beyotime (China). The LDH assay kit was sourced from Nanjing Jiancheng Bioengineering Institute (China). Propidium iodide solution was obtained from Meilunbio (China). The Prime Script-RT kit, SYBR Premix Ex Taq, miRNA first Strand cDNA Synthesis Kit, and SYBR qPCR Master Mix were obtained from Vazyme Biotech (China). TRIzol reagent was obtained from Thermo Fisher Scientific (United States). TNF-α, IL-4, IL-10, and IL-1β ELISA kits, as well as TGF-β1 and β-actin antibodies, were obtained from Proteintech (United States). Antibodies against CD14, CD29, CD34, CD44, CD45, CD73, CD90, CD105, and CD11b were obtained from eBioscience (United States). micrON rno-miR-199a-3p mimic, micrON mimic negative control (NC), and micrON hsa-miR-199a-3p mimic were obtained from RiboBio (China).

### 2.2 Composition of BSHXF

The BSHXF formulation consists of the following medicinal herbs: *Eucommia ulmoides* Oliv (15 g), *Cullen corylifolium* (10 g), *Achyranthes bidentata* Blume (10 g), *Salvia miltiorrhiza* Bunge (12 g), *Clematis chinensis* Osbeck (10 g), and *Chaenomeles speciosa* Nakai (9 g). All botanical specimens were sourced and pharmacognostically validated through the institutional review board of Hunan University of Chinese Medicine First Hospital, adhering to the Chinese Pharmacopoeia (2015 edition) quality protocols. Post-certification materials underwent standardized storage protocols at the university’s Medical Innovation Core Facility. After precise weighing according to the specified ratios, the herbs underwent three rounds of water decoction and reflux extraction: the first cycle used eight times the volume of water for 1 h, and the second and third cycles used six times the volume of water for 1 h each. The combined extracts were filtered and concentrated to 3 g/mL using a rotary evaporator.

### 2.3 *In Vitro* experiments

#### 2.3.1 Preparation of BSHXF drug-containing serum

Thirty Sprague-Dawley male rats (200 ± 10 g) underwent 7-day environmental acclimation in HUCM’s SPF-level animal facility (ambient temperature 22°C ± 1°C, 12 h light/dark cycle) with *ad libitum* access to standard chow. Following randomization via computer-generated sequence (n = 15/cohort), experimental arms comprised: 1) BSHXF-treated serum cohort receiving 3×HED (human equivalent dose, calculated by body surface area normalization for 60 kg adult), 2) vehicle control administered equivalent saline volumes. Twice-daily oral gavage continued for seven consecutive days. Terminal blood collection proceeded 60 min post-final dosing under aseptic pentobarbital anesthesia (0.2%, 3 mL/kg i. p.). Aortic blood underwent biphasic processing: primary centrifugation (4°C, 3000g, 20 min) followed by serum heat inactivation (56°C/30 min), microfiltration (0.22 μm), deionization, and cryopreservation (−20°C). Protocol compliance was verified by HUCM Institutional Animal Care Committee (IACUC No. LLBH-202111250003).

#### 2.3.2 Isolation, culture, and identification of ADSCs

Human ADSCs were obtained through enzymatic digestion of adipose tissue acquired via liposuction procedures performed on healthy female donors (20–35 years old) during cosmetic surgeries conducted in the Plastic Surgery Department of Hunan University of Chinese Medicine First Affiliated Hospital. Specimens were collected intraoperatively with informed consent. The study adhered to the ethical principles of the Declaration of Helsinki and Tokyo Declaration and was approved by the Medical Research Ethics Committee of the First Affiliated Hospital of Hunan University of Chinese Medicine (Ethical Approval No.: HN-LL-KY-2022-033-01). Written informed consent was obtained from all participants. ADSCs were isolated and cultured as previously described (Ai et al., 2023). Briefly, adipose tissue was rinsed thoroughly with PBS containing penicillin-streptomycin, minced into 1 mm^3^ fragments, and digested with 0.2% collagenase at 37°C for 60 min. Following centrifugation (1,000 rpm, 5 min), the cellular pellet underwent resuspension in complete DMEM containing 10% fetal bovine serum and 1% penicillin-streptomycin. Cells were plated into culture vessels and incubated under standard conditions (37°C, 5% CO_2_, humidified atmosphere). Third-passage ADSCs were used for subsequent experiments. Human ADSCs were analyzed for surface markers using flow cytometry, as previously described by Megaloikonomos (Palumbo et al., 2018).

#### 2.3.3 Cell transfection

Exponentially growing ADSCs were plated in 6-well plates at 5 × 10^6^ cells per well. After stabilization, human ADSCs were transfected with either the micrON hsa-miR-199a-3p mimic or the corresponding mimic negative control following the manufacturer’s protocol. Following 24-h culture under standard conditions (37°C, 5% CO_2_), transfection efficiency was assessed.

#### 2.3.4 Establishment of cellular models and experimental grouping

In cellular experiments, T-BHP induces oxidative stress-mediated cellular senescence, mimicking the degenerative microenvironment of intervertebral discs. Based on previous studies (Duan et al., 2023), we established an ADSCs senescence model using T-BHP-induced oxidative damage. ADSCs were treated with T-BHP at concentrations of 0, 25, 50, 100, 250, and 500 μmol/L. Following a 24-h incubation period, the half-maximal inhibitory concentration (IC50) was assessed using CCK-8 assay to identify the optimal modeling concentration. ADSCs were exposed to BSHXF drug-containing serum at varying concentrations (0%, 5%, 10%, 20%, 30%, 40%), and cell viability was evaluated using the CCK-8 assay at 24, 48, and 72 h. The experimental groups were designated as follows: Normal serum control group: non-medicated serum; Model control group: T-BHP + non-medicated serum; Drug-containing serum group: T-BHP + BSHXF drug-containing serum; miR-199a-3p overexpression group: miR-199a-3p mimic-transfected + T-BHP + BSHXF drug-containing serum; miR-199a-3p empty vector group: miR-NC-transfected (negative control) + T-BHP + non-medicated serum.

#### 2.3.5 CCK-8 assay for cell viability

Exponentially growing cells were trypsinized, counted, and plated in 96-well plates at 5 × 10^3^ cells/well with 100 μL culture medium per well. Each experimental group had wells set up in triplicate. Following cell attachment, each well received 10 μL of CCK-8 reagent diluted in complete culture medium. The drug-containing medium was aspirated, and 100 μL of CCK-8-containing medium was added to each well. Cells were incubated at 37°C in a 5% CO_2_ atmosphere for 2 h. Optical density (OD) values were measured at 450 nm using a microplate reader.

#### 2.3.6 Flow cytometry for cell cycle analysis

Cells in exponential growth were collected, resuspended in 1 mL of ice-cold PBS, and centrifuged at 800 rpm for 5 min. The supernatant was carefully aspirated. Subsequently, cells were gently resuspended in 400 μL PBS to achieve single-cell suspension. Ice-cold 100% ethanol (1.2 mL) was added dropwise while vortexing, adjusting the final ethanol concentration to 75%. Cells were preserved at 4°C overnight. After fixation, samples were centrifuged at 800 rpm for 5 min, followed by discarding the supernatant. Cells were washed twice with 1 mL of ice-cold PBS to eliminate residual ethanol, with centrifugation performed after each wash. Cells were incubated with 150 μL of propidium iodide solution at 4°C for 30 min, protected from light. Stained cells were analyzed in flow cytometry tubes using a flow cytometer with a 488 nm argon-ion laser for PI excitation and a 630 nm bandpass filter for fluorescence detection. Each sample was analyzed by acquiring 10,000 events using FSC/SSC dot plots. Doublet exclusion gating strategy was applied to eliminate cell aggregates and debris. Cell cycle phase distribution was quantified based on PI fluorescence intensity histograms.

#### 2.3.7 SA-β-gal detection method for cellular senescence

Following medium aspiration, the cells underwent PBS washing. Cells were fixed at room temperature for 15 min using 1 mL of β-galactosidase staining fixative. The fixative was removed, followed by three washes with PBS, each lasting 3 min. Following PBS aspiration, 1 mL of staining solution was introduced into each well. Cells were incubated at 37°C for 24 h with the 6-well plate sealed using parafilm to minimize evaporation. SA-β-Gal-positive cells (blue staining) were visualized and photographed under a standard optical microscope. Three random fields per group were analyzed for quantitative assessment.

#### 2.3.8 LDH activity assay

Treated ADSCs from each group were plated in 6-well plates (5 × 10^5^ cells/well) and maintained at 37°C for 24 h. After collecting the supernatant, processing was conducted following the manufacturer’s instructions provided with the LDH assay. Absorbance at 450 nm was determined by a microplate reader, and LDH release levels in all groups were subsequently quantified.

#### 2.3.9 Immunofluorescence double staining for CD73 and COL2A1 Co-Expression

Following three PBS washes, cell culture slides were processed through 4% paraformaldehyde fixation (30 min) and subsequent triple PBS rinses (5 min each). Cells were treated with 0.3% Triton X-100 at 37°C for 30 min to permeabilize them, followed by three washes with PBS. Non-specific binding was blocked with 5% BSA-PBS at room temperature for 60 min. Primary antibodies targeting CD73 were diluted to optimal concentrations followed by overnight incubation at 4°C. Unbound antibodies were removed by three PBS washes. HRP-conjugated secondary antibodies, matched to the species of the CD73 primary antibody, were incubated in the dark at room temperature for 30 min, followed by three washes with PBS. Tyramide signal amplification using TSA-520 fluorescent dye was conducted for 10 min, followed by three PBS washes. Antibody stripping was conducted using pre-warmed (37°C) stripping buffer. Samples underwent two 10-min incubations, followed by three PBS washes. Endogenous peroxidase activity was blocked with a peroxidase inhibitor through 10-min dark incubation at room temperature, succeeded by triplicate PBS rinses. Following a 60-min re-blocking with 5% BSA-PBS, primary antibodies targeting COL2A1 were applied at optimal dilutions and incubated overnight at 4°C.Slides underwent three PBS washes. HRP-conjugated secondary antibodies targeting COL2A1 underwent 30-min ambient dark incubation, with subsequent triplicate PBS rinses completing the procedure. TSA-570 fluorescent dye was employed for a 10-min signal amplification, followed by three PBS washes of the slides. A second antibody stripping was conducted, followed by three PBS washes. Nuclei were counterstained with DAPI at room temperature for 10 min in the dark, followed by three PBS rinses. Slides were mounted in an anti-fade glycerol medium and imaged with a fluorescence microscope using suitable filters. Images were captured and analyzed for co-localization of CD73 and COL2A1.

#### 2.3.10 Flow cytometry for intracellular ROS measurement

A 10 μM DCFH-DA probe was generated through 1,000-fold dilution of the stock solution (10 mM) in serum-free medium. Following aspiration of the original medium from treated cells, the prepared probe was applied to ensure complete coverage of the cellular monolayer. Cells were incubated at 37°C in a humidified CO_2_ environment for 20 min to enhance DCFH-DA uptake. Following incubation, cells were rinsed three times with serum-free medium to eliminate extracellular DCFH-DA. Cells were trypsinized and harvested by centrifugation. Intracellular ROS levels were quantified using a flow cytometer.

#### 2.3.11 ELISA for inflammatory cytokine expression (TNF-α, IL-1β, IL-4, IL-10)

Reagents underwent ambient acclimation (18°C–25°C) for ≥30 min prior to being processed per the manufacturer’s protocol. Standard and sample wells were designated. Each well was loaded with 100 μL of standard or test sample. Following gentle homogenizing agitation, the plate was sealed and maintained at 37°C for 2-h incubation. The solution was aspirated, and the plate was washed four times with 300 μL of wash buffer per well (30 s per wash), followed by thorough drying. Subsequently, 100 μL of detection antibody working solution was dispensed into all wells. Following resealing, the plate underwent 1-h incubation at 37°C. Upon completion of aspiration, four cycles of washing were performed using 300 μL wash buffer (30 s per cycle) prior to thorough drying. Next, 100 μL of HRP-conjugated streptavidin working solution was added to each well. The plate was sealed and incubated at 37°C for 40 min. After incubation, the solution was discarded, and the plate underwent four additional washes with 300 μL of wash buffer (30 s per wash), followed by drying. A volume of 100 μL substrate solution was added to each well, and the plate was incubated in the dark at 37°C for 15–20 min for chromogenic reaction. The reaction was terminated by adding 100 μL stop solution to each well. Within 5 min of termination, OD values were determined at 450 nm via a microplate reader, with cytokine concentrations derived by interpolating sample readings against the standard curve prepared from serial dilutions.

#### 2.3.12 RT-qPCR for miR-199a-3p expression analysis

Total RNA was extracted from cells using the TRIzol reagent. To quantify mRNA expression levels, RNA was reverse-transcribed into complementary DNA (cDNA) using the PrimeScript RT Kit according to the manufacturer’s protocol. cDNA amplification was performed with SYBR Premix Ex Taq. For miR-199a-3p quantification, RNA was reverse-transcribed using the miRNA first Strand cDNA Synthesis Kit (via stem-loop priming), followed by amplification with the miRNA Universal SYBR qPCR Master Mix. The relative expression levels of target genes were calculated using the 2^−ΔΔCT^ method. Primer sequences were designed by retrieving target gene sequences from NCBI and using Primer5 software. All primers were synthesized by Tsingke Biotechnology (Beijing, China). Table 2 lists the primer sequences for the target and reference genes used in this study.

**TABLE 2:** The primers used in RT-qPCR.

Gene name	Primer sequence (5’→3′)
Rno-miR-199a-3p	F:ACAGTAGTCTGCACATTGGTTA
	R:GCTGTCAACGATACGCTACGTA
Has-miR-199a-3p	F:CCCAGTGTTCAGACTACCTGTTC
	R:GCTGTCAACGATACGCTACGTA
H-U6	F:CTCGCTTCGGCAGCACA
	R:AACGCTTCACGAATTTGCGT
R-5S	F:GCCTACAGCCATACCACCCGGAA
	R:CCTACAGCACCCGGTATCCCA

#### 2.3.13 Western blot analysis of COL2A1, SOX9, ACAN, TGF-β1, Smad2, Smad3, P-Smad2, and P-Smad3 expression

After washing with ice-cold PBS, cells were lysed with 200 μL of LRIPA buffer. Cells were collected using a scraper and subjected to 1.5 min of sonication. Following a 10-min ice incubation, the lysate was centrifuged at 12,000 rpm for 15 min (4°C) with a pre-cooled centrifuge. Protein quantification was performed with a BCA assay kit according to the manufacturer’s protocol. Separation gels were prepared by gentle agitation and immediately poured after TEMED addition, followed by sealing with isopropanol. Stacking gels were prepared to fill the remaining space and allowed to polymerize. Protein samples (20 μL supernatant) were mixed with 5× loading buffer (30 μL), heated for 5 min, and then rapidly cooled on ice. Proteins were transferred to PVDF membranes post-electrophoresis and blocked with 5% non-fat milk in 1× PBST at room temperature for 90 min. Membranes were incubated overnight at 4°C with primary antibodies diluted in 1× PBST: anti-P-Smad3 (1:2000), anti-COL2A1 (1:500), anti-Smad2 (1:500), anti-Smad3 (1:500), anti-P-Smad2 (1:500), anti-SOX9 (1:10,000), anti-ACAN (1:1,000), anti-TGF-β1 (1:1,000), and anti-β-actin (1:5000). The next day, membranes were subjected to a 90-min room temperature incubation with HRP-linked secondary antibodies, including both goat-derived anti-mouse and anti-rabbit IgG (H + L) antibodies, all diluted 1:5000 in 1× PBST. Protein bands were visualized with ECL detection reagents, and their intensities quantified using Bio-Rad’s QuantityOne software.

### 2.4 *In Vivo* experiments

#### 2.4.1 Isolation, culture, and identification of ADSCs

ADSCs were isolated, cultured, and characterized from SD rats following previously reported protocols (Megaloikonomos et al., 2018). Briefly, rats were euthanized via anesthetic overdose, and the inguinal fat pads were aseptically excised. Primary ADSCs were obtained through sequential enzymatic digestion. The isolated cells were seeded in culture flasks and maintained at 37°C in a humidified 5% CO_2_ incubator. Third-passage ADSCs were utilized for subsequent experiments. Rat ADSCs were phenotypically characterized by flow cytometry for surface markers, as described in prior studies.

#### 2.4.2 Cell transfection

ADSCs at log-phase were plated in 6-well plates (5 × 10^6^ cells/well). After achieving stable growth, rat ADSCs underwent transfection with micrON™ rno-miR-199a-3p mimic or its negative control counterpart (NC) following manufacturer guidelines. Cultures were kept under standard conditions (37°C, 5% CO_2_) for 24 h, after which transfection efficiency was assessed.

#### 2.4.3 Animal model establishment and experimental grouping

An IDD model was established in SD rats via percutaneous needle puncture of the caudal intervertebral discs (Co5/6, Co6/7, Co7/8), as previously described (Wang et al., 2019). Following intraperitoneal administration of 0.2% sodium pentobarbital (3 mL per kilogram body weight), rats were anesthetized and placed in dorsal recumbency. The tail was disinfected with povidone-iodine after PBS washing. Under fluoroscopic guidance, a 25G needle was advanced orthogonally to the skin surface and aligned with the cartilage endplate to reach the central region of the target discs, avoiding caudal blood vessels. The needle was rotated 180° and held for 5 s before removal. Postoperatively, the tail was disinfected again, and rats were placed prone in cages with 12-h fasting to prevent ileus. At week two post-modeling, transfected ADSCs were injected into the Co5/6, Co6/7, and Co7/8 discs. Briefly, anesthetized rats were disinfected, and 20 μL of ADSCs (1.0 × 10^6^ cells/mL in culture medium, total 2 × 10^4^ cells/disc) were injected per disc using a 31G needle-equipped 50 μL microsyringe. The injection site was compressed for 1 min to prevent leakage.

Thirty-six male SD rats (2–4 months old, 200–300 g) were randomly assigned to six groups (n = 6 per group):Blank control group: Normal diet + oral gavage of distilled water; Model group: IDD model + oral gavage of distilled water; miR-199a-3p overexpression group: IDD model + intradiscal injection of miR-199a-3p-overexpressing ADSCs; miR-199a-3p overexpression + BSHXF group: IDD model + intradiscal miR-199a-3p-overexpressing ADSCs + oral gavage of BSHXF (5.94 g/kg); miR-199a-3p empty vector group: IDD model + intradiscal injection of miR-199a-3p empty vector-transfected ADSCs; miR-199a-3p empty vector + BSHXF group: IDD model + intradiscal empty vector-transfected ADSCs + oral gavage of BSHXF (5.94 g/kg). All six groups underwent oral gavage administration for 4 weeks. Tissue samples were collected after this period for subsequent assessment of relevant parameters. In preliminary experiments (Duan et al., 2023), six major compounds in BSHXF extract were identified via UPLC-Q-TOF-MS: geniposidic acid, psoralen, isopsoralen, psoralenoside, isopsoralenoside, and tanshinone IIA.

#### 2.4.4 Histological evaluation

Intervertebral disc tissues were fixed in 4% paraformaldehyde for 48 h and decalcified in neutral 10% (v/v) EDTA solution for 20 days. Tissues were paraffin-embedded, sagittally sectioned at 5 μm, and affixed onto glass slides; histological assessment through Hematoxylin-eosin and Safranin O-Fast Green staining was conducted to analyze disc structural characteristics. Histopathological changes were graded using a validated scoring system (5–15 points), where higher scores indicate severe degeneration (Han et al., 2008).

#### 2.4.5 IHC analysis

Tissue samples underwent fixation in 10% neutral-buffered formalin before being embedded in paraffin and sliced into 5 μm sections. After mounting on adhesive-coated slides, specimens were dried at 60°C for 2 h, subjected to three xylene washes for deparaffinization, and rehydrated through a descending ethanol series. For antigen retrieval, slides were treated with 30 μL urea solution during 37°C incubation (30 min) and subsequently rinsed thrice with PBS (3-min intervals). Endogenous peroxidase blockade was achieved using 1% periodic acid (15 min, room temperature). Primary antibodies targeting collagen types I (Col1A1, 1:200) and II (Col2A1, 1:200) were applied for overnight incubation at 4°C. This was followed by 37°C treatment with HRP-conjugated anti-rabbit IgG polymer (1:200, 30 min). Chromogenic development employed DAB substrate prior to hematoxylin counterstaining. Finally, sections underwent sequential ethanol dehydration, xylene clearance, and neutral balsam mounting. Digital slide scanning captured images, with collagen-positive area quantification through integrated optical density (IOD) measurements performed with Image-Pro Plus 6.0 software (Media Cybernetics, United States).

#### 2.4.6 RT-qPCR for miR-199a-3p expression analysis

The experimental methods and procedures were identical to those described in Section 2.3.12.

#### 2.4.7 Western blot analysis of TGF-β1, Smad2, Smad3, P-Smad2, and P-Smad3 expression

Intervertebral disc tissue proteins underwent RIPA buffer lysis, with subsequent protein concentration determination via BCA assay. Following a 10-min centrifugation at 12,000 × g, the extracted proteins underwent denaturation in boiling water for an equivalent duration. Proteins were separated by 10% SDS-PAGE at 120 mV and subsequently transferred to PVDF membranes at 200 mA for approximately 1 h. The membranes were blocked with 5% skim milk and 4% BSA at 25°C for 2 h, followed by overnight incubation at 4°C with the following primary antibodies: TGF-β1 (1:1,000), Smad2 (1:2000), P-Smad2 (1:1,000), Smad3 (1:2000), P-Smad3 (1:2000), and β-actin (1:5000). HRP-conjugated secondary antibodies, including Goat anti-Mouse IgG (H + L) (1:5000) and Goat anti-Rabbit IgG (H + L) (1:5000), were diluted in 1× PBST and incubated with the membranes at room temperature for 90 min. Following membrane incubation, triple washing cycles with 1× PBST were conducted (10-min intervals), succeeded by chemiluminescent detection of protein bands and subsequent quantitative assessment through Quantity One software.

### 2.5 Statistical analysis

Statistical processing was conducted with SPSS 23 (IBM Corp., Armonk, NY, United States). Parametric continuous variables underwent Student’s t-test evaluation, whereas nonparametric or categorical datasets received chi-square analysis or Wilcoxon rank-sum method application where applicable. Statistical significance threshold was established at p < 0.05.

## 3 Results

### 3.1 Characterization of ADSCs surface markers

**FIGURE 1:**
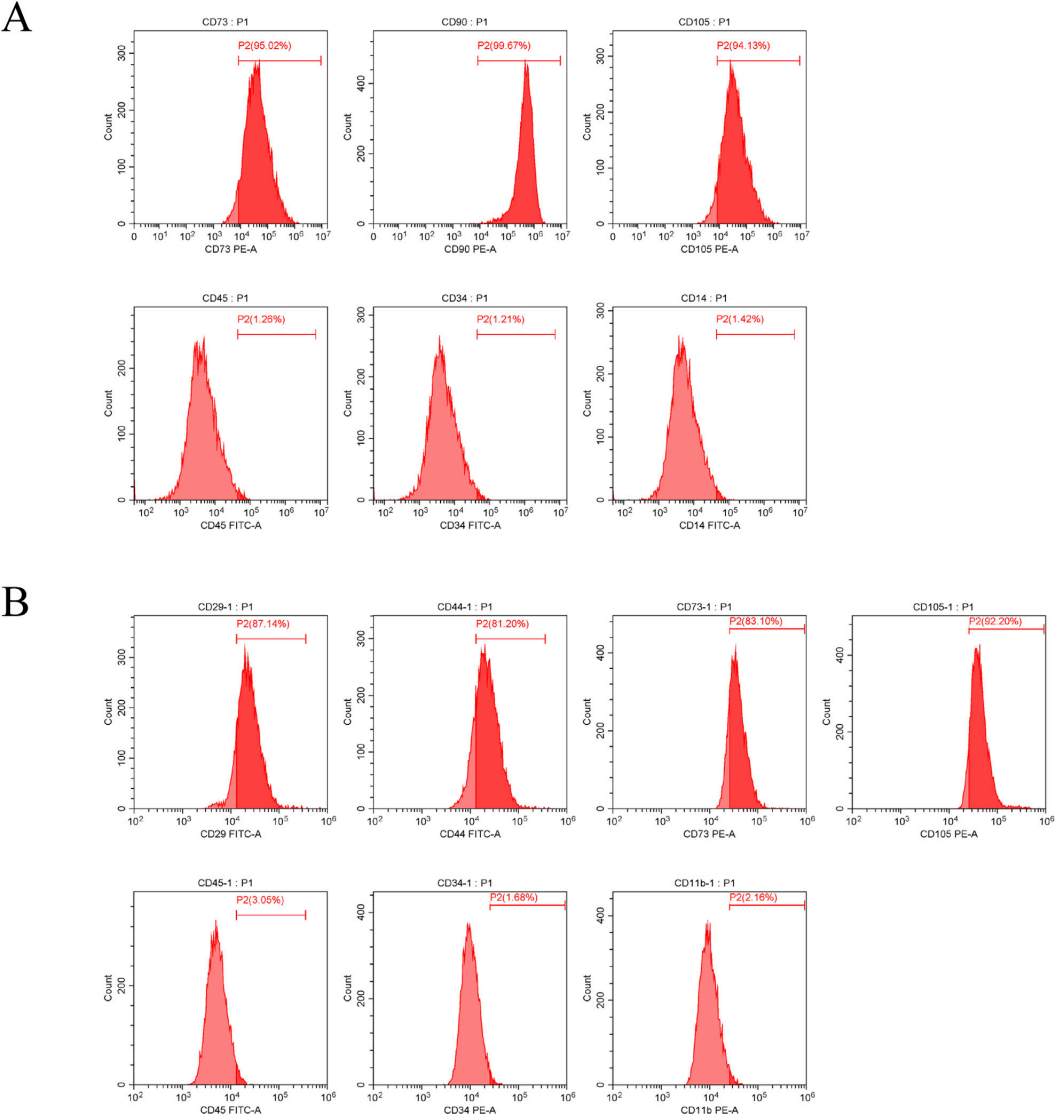
**(A)** Identification of surface markers on human ADSCs; **(B)** Identification of surface markers on rat ADSCs.

To confirm that the ADSCs used in this study met internationally recognized biological standards (Dominici et al., 2006), we systematically characterized cell surface markers via flow cytometry, following previously reported methodologies (Megaloikonomos et al., 2018; Palumbo et al., 2018). For human ADSCs, mesenchymal stem cell-specific markers (CD73: 95.02%, CD90: 99.67%, CD105: 94.13%) exhibited high expression, whereas hematopoietic/non-mesenchymal markers (CD45: 1.26%, CD34: 1.21%, CD14: 1.42%) showed negligible expression. In rat ADSCs, high expression was observed for mesenchymal markers (CD29: 87.14%, CD44: 81.20%, CD73: 83.10%, CD105: 92.20%), while hematopoietic/non-mesenchymal markers (CD45: 3.05%, CD34: 1.68%, CD11b: 2.16%) demonstrated minimal expression. These data collectively indicate that the isolated ADSCs displayed canonical mesenchymal stem cell phenotypic characteristics and met purity criteria for subsequent experimental applications (Figure 1).

### 3.2 Validation of ADSCs transfection efficiency

**FIGURE 2:**
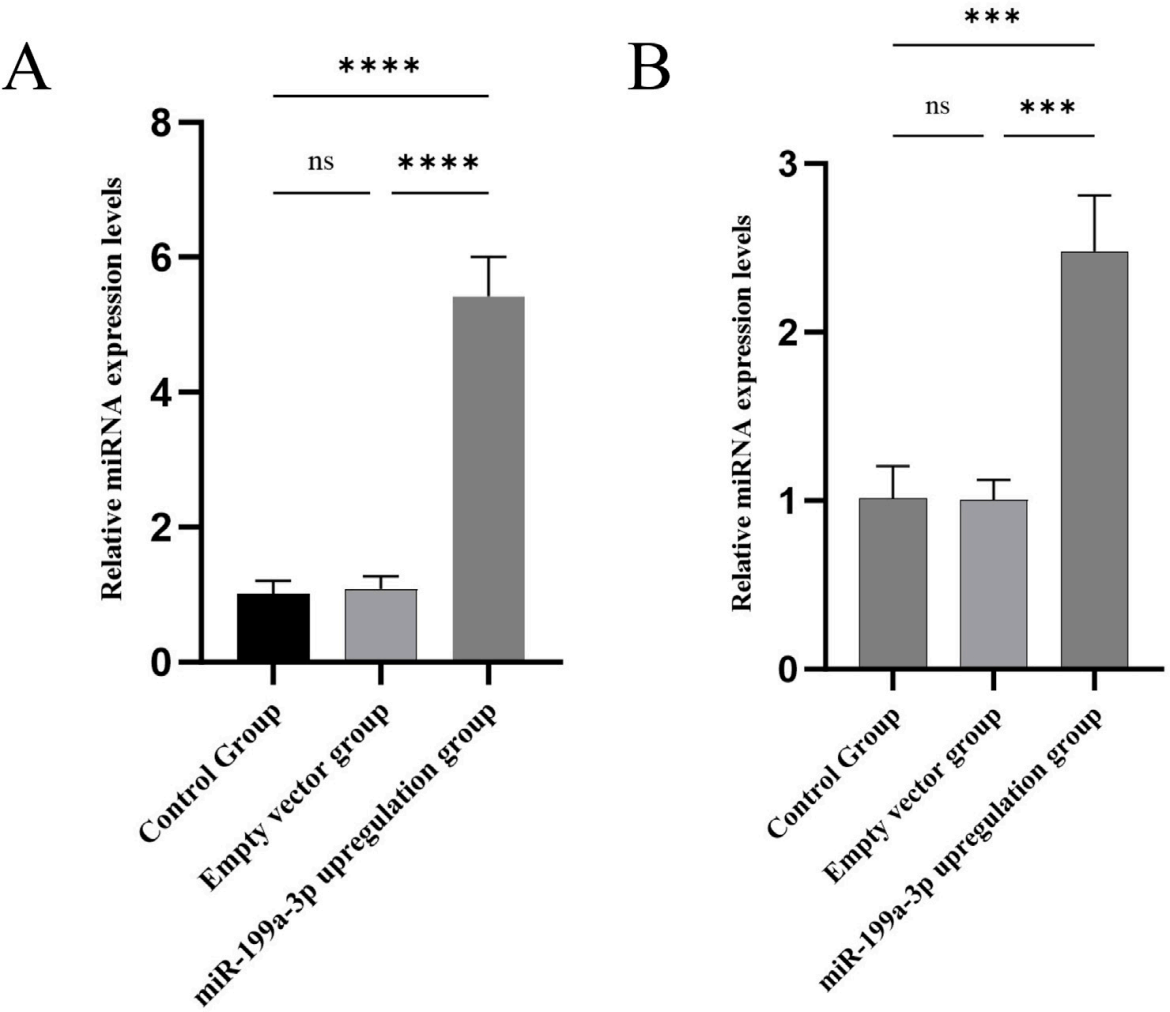
**(A)** RT-qPCR analysis of relative expression levels of miR-199a-3p in each experimental group of *in vitro* cell assays; **(B)** RT-qPCR analysis of relative expression levels of miR-199a-3p in each experimental group of *In vivo* animal models. Control Group: Cells were cultured under normal conditions without any treatment. Empty vector group: Cells were transfected with micrON mimic NC. miR-199a-3p upregulation group: Cells were transfected with miR-199a-3p mimic. nsP >0.05; *p < 0.05; **p < 0.01; ***p < 0.001; ****p < 0.0001.

To validate the transfection efficiency in ADSCs, the relative expression of miR-199a-3p was assessed via RT-qPCR in both cellular and animal experiments. The results (Figure 2) demonstrated that: (1) Compared to the Control Group (cells cultured under standard conditions without treatment) and the Empty vector group (cells transfected with miR-199a-3p mimic negative control, NC), no significant increase in miR-199a-3p expression was observed, confirming that the empty vector transfection did not induce non-specific miRNA expression changes. This validated the reliability of the negative control. (2) In contrast, the miR-199a-3p upregulation group (cells transfected with miR-199a-3p mimic) exhibited a marked elevation in miR-199a-3p expression compared to the Control Group, indicating successful overexpression of miR-199a-3p in transfected ADSCs. These findings confirmed the transfection efficacy and established a reliable foundation for subsequent experiments.

### 3.3 Screening of modeling conditions and effective concentration of drug-containing serum

**FIGURE 3:**
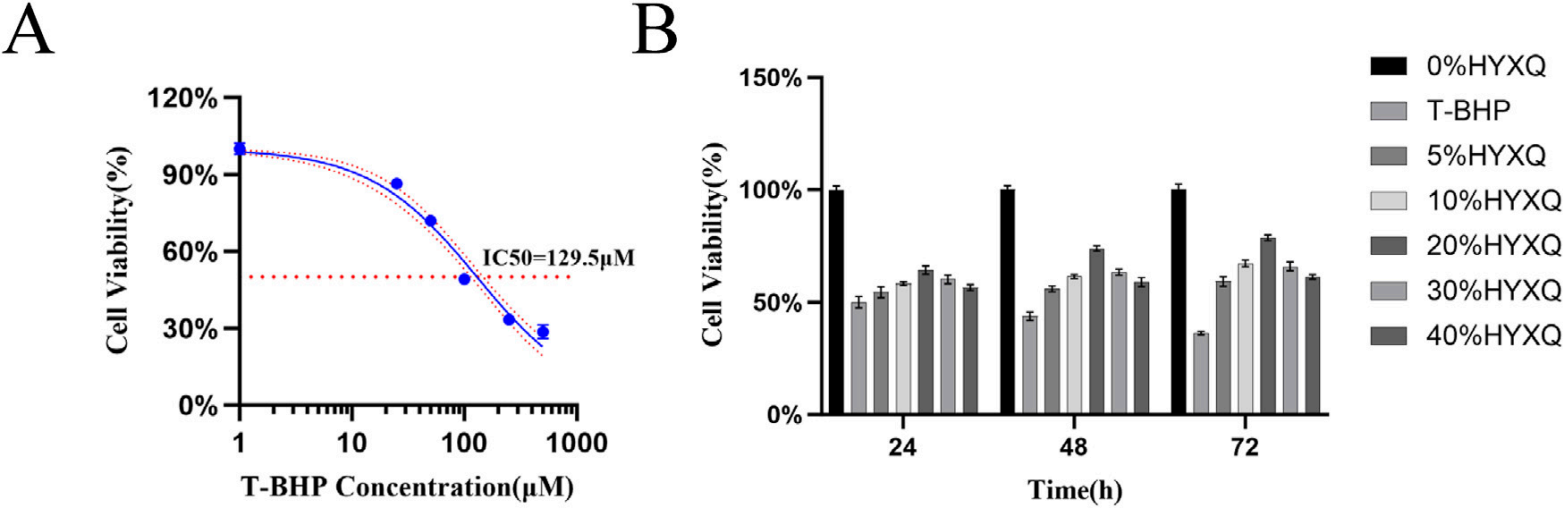
**(A)** The IC50 concentration of T-BHP on ADSCs; **(B)** The protective effect of BSHXF-containing serum at varying concentrations (24, 48, and 72 h) on 130 μM T-BHP-induced senescence of ADSCs; **(C)** Assessment of cell viability in each experimental group. nsP >0.05; *p < 0.05; **p < 0.01; ***p < 0.001; ****p < 0.0001.

The impact of T-BHP on ADSCs proliferation was evaluated at concentrations of 0, 25, 50, 100, 250, and 500 μM using CCK-8 assays after 24 h. Cytotoxicity was determined, yielding a calculated IC50 value of 130 μM (Figure 3A). The effect of varying concentrations (0%, 5%, 10%, 20%, 30%, 40%) of drug-containing serum on ADSCs proliferation was assessed using CCK-8 assays at 24, 48, and 72 h, with cell viability subsequently calculated. The results indicated that 20% drug-containing serum was the optimal concentration after 72 h of culture (Figure 3B).

### 3.4 ADSCs proliferation and damage

**FIGURE 4:**
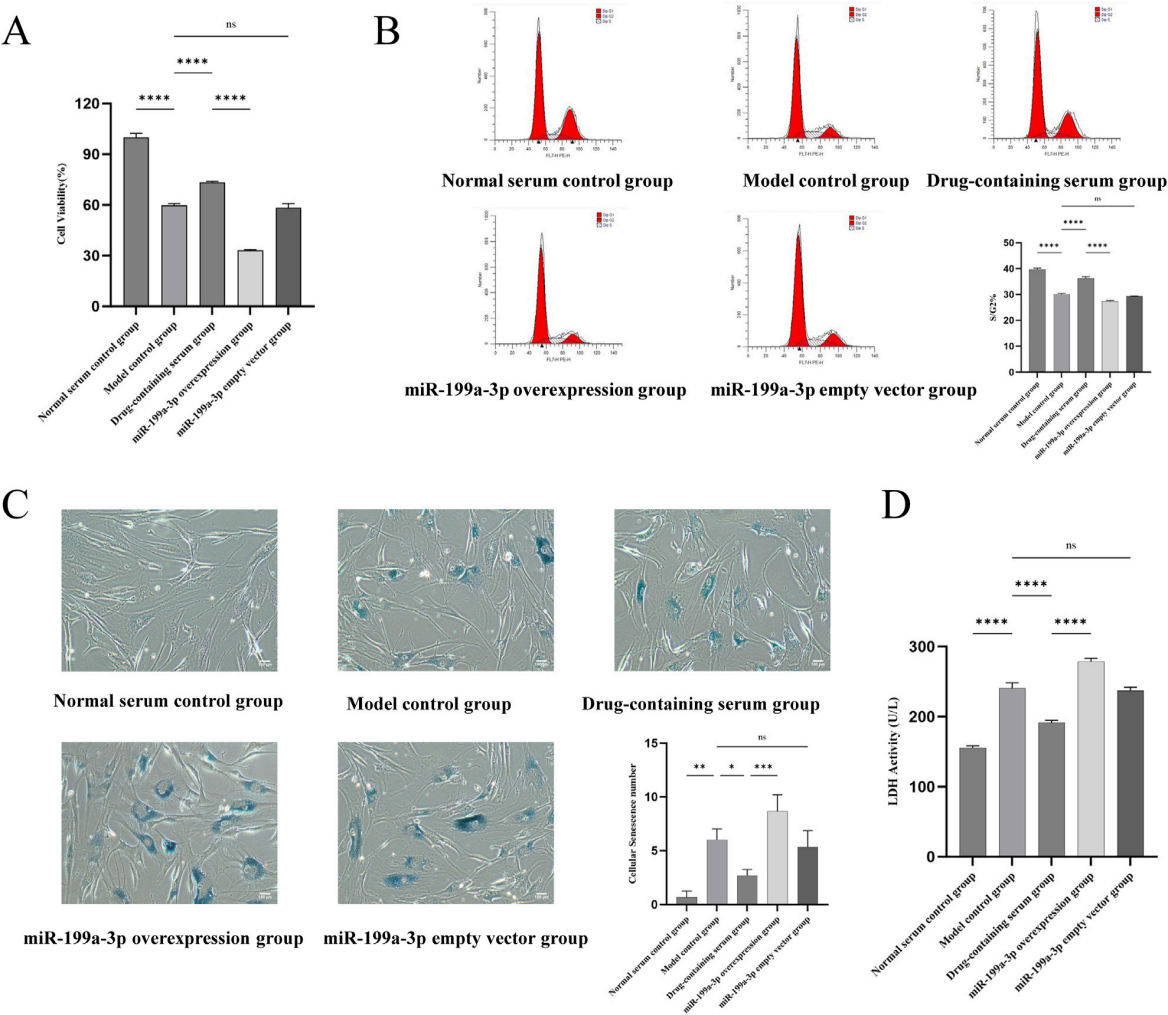
**(A)** Results of the CCK-8 assay assessing cell viability in each experimental group; **(B)** Flow cytometric analysis of cell cycle distribution; **(C)** Analysis of SA-β-Gal staining results; **(D)** Measurement of lactate dehydrogenase activity. nsP >0.05; *p < 0.05; **p < 0.01; ***p < 0.001; ****p < 0.0001.

In the ADSCs proliferation assay, the CCK-8 assay was employed to evaluate the proliferative activity of cells in different treatment groups. The results revealed that the cell viability in the Model control group was significantly reduced compared to the Normal serum control group, indicating that T-BHP-induced oxidative stress markedly impaired cellular activity. The cell viability in the Drug-containing serum group was notably higher than that in the Model control group, suggesting that BSHXF could partially alleviate T-BHP-induced cellular damage. The miR-199a-3p overexpression group exhibited the lowest cell viability, which was significantly lower than that of the Drug-containing serum group, demonstrating that miR-199a-3p overexpression further inhibited cellular proliferation (Figure 4A). Analysis of the ADSCs cell cycle showed that the proportion of cells in the S/G2 phase was significantly decreased in the Model control group compared to the Normal serum control group. This result demonstrated that T-BHP-induced oxidative stress substantially inhibited cell proliferation by arresting the cell cycle at the G1 phase, thereby reducing the proportion of cells entering the S/G2 phase. The Drug-containing serum group displayed a restored proportion of S/G2-phase cells, which was significantly higher than that of the Model control group. This finding indicates that BSHXF alleviated T-BHP-induced cell cycle arrest, promoted the normal transition from the G1 to S/G2 phase, and restored cellular proliferation capacity. The miR-199a-3p overexpression group exhibited the lowest proportion of S/G2-phase cells, which was significantly lower than that of the Drug-containing serum group. This observation indicates that miR-199a-3p overexpression exacerbated T-BHP-induced cell cycle arrest, suppressed the ability of cells to enter the S/G2 phase, and diminished proliferation levels (Figure 4B).

SA-β-Gal staining results revealed that the number of senescent cells in the Model control group was significantly increased compared to the Normal serum control group, indicating that T-BHP-induced oxidative stress markedly exacerbated cellular senescence. In contrast, the Drug-containing serum group exhibited a notable reduction in senescent cells compared to the Model control group, demonstrating that BSHXF attenuated T-BHP-induced oxidative stress and effectively suppressed senescence progression. The miR-199a-3p overexpression group displayed the highest number of senescent cells, suggesting that miR-199a-3p overexpression further aggravated cellular senescence (Figure 4C). Analysis of LDH leakage rates across groups showed that LDH activity in the Model control group was significantly elevated compared to the Normal serum control group. The increased LDH release reflected substantial membrane damage caused by T-BHP-induced oxidative stress, with heightened cell membrane permeability indicating cellular injury. The Drug-containing serum group exhibited a significant decrease in LDH activity compared to the Model control group, indicating that BSHXF partially mitigated T-BHP-induced oxidative stress, restored membrane integrity, and exerted protective effects. Conversely, the miR-199a-3p overexpression group displayed the highest LDH activity, demonstrating that miR-199a-3p overexpression caused greater disruption of membrane integrity and exacerbated cellular damage (Figure 4D).

### 3.5 Nucleus pulposus-like differentiation and ECM synthesis capacity of ADSCs

**FIGURE 5:**
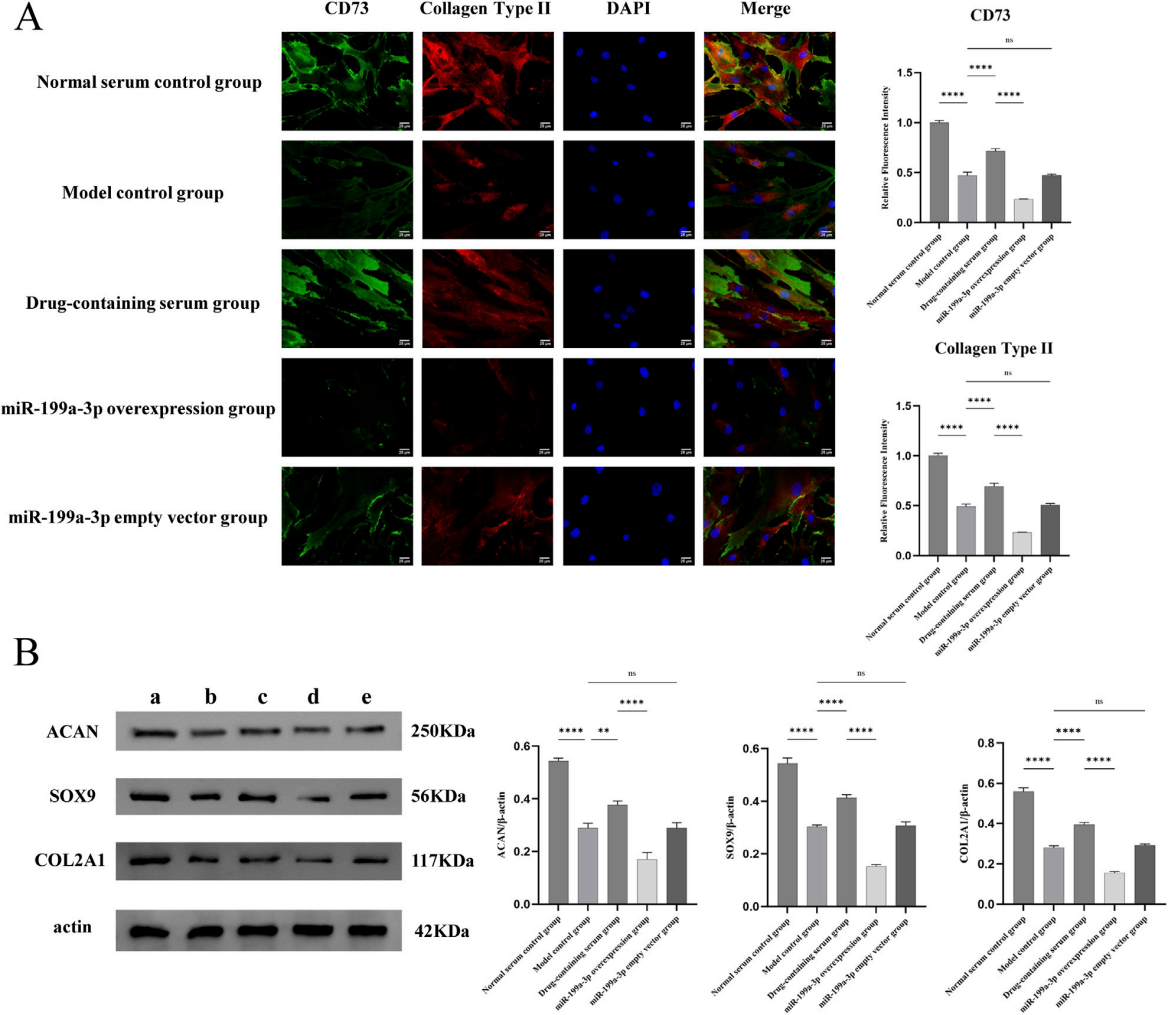
**(A)** Immunofluorescence double staining analysis for the co-expression of CD73 and type II collagen; **(B)** Evaluation of the extracellular matrix synthesis capacity of ADSCs. **(A)** Normal serum control group; **(B)** Model control group; **(C)** Drug-containing serum group; **(D)** miR-199a-3p overexpression group; **(E)** miR-199a-3p empty vector group. nsP >0.05; *p < 0.05; **p < 0.01; ***p < 0.001; ****p < 0.0001.

The nucleus pulposus-like differentiation capacity of ADSCs was assessed using immunofluorescence double staining for co-expression of CD73 and COL2A1. The results demonstrated that the expression of CD73 and COL2A1 in the Model control group was significantly decreased compared to the Normal serum control group, indicating that T-BHP-induced oxidative stress markedly suppressed the nucleus pulposus-like differentiation potential of ADSCs. In contrast, the Drug-containing serum group exhibited a significant upregulation of CD73 and COL2A1 expression compared to the Model control group, suggesting that BSHXF partially restored the nucleus pulposus-like differentiation capacity of ADSCs under T-BHP-induced damage. Conversely, the miR-199a-3p overexpression group showed a pronounced reduction in CD73 and COL2A1 expression levels, demonstrating that miR-199a-3p overexpression impaired the nucleus pulposus-like differentiation ability of ADSCs (Figure 5A).

The ECM synthesis capacity was evaluated by Western blot analysis of COL2A1, SOX9, and ACAN expression levels. In the Model control group, the expression levels of COL2A1, SOX9, and ACAN were significantly reduced compared to the Normal serum control group, indicating that T-BHP-induced oxidative stress markedly suppressed the ECM synthesis capacity of ADSCs. Conversely, the Drug-containing serum group exhibited a significant restoration of COL2A1, SOX9, and ACAN expression levels compared to the Model control group, demonstrating that BSHXF effectively alleviated T-BHP-induced oxidative stress and partially recovered the ECM synthesis capacity of ADSCs. The miR-199a-3p overexpression group displayed a further decrease in COL2A1, SOX9, and ACAN expression levels, suggesting that miR-199a-3p overexpression exerted a pronounced inhibitory effect on ECM synthesis (Figure 5B).

### 3.6 Assessment of cellular microenvironment

**FIGURE 6:**
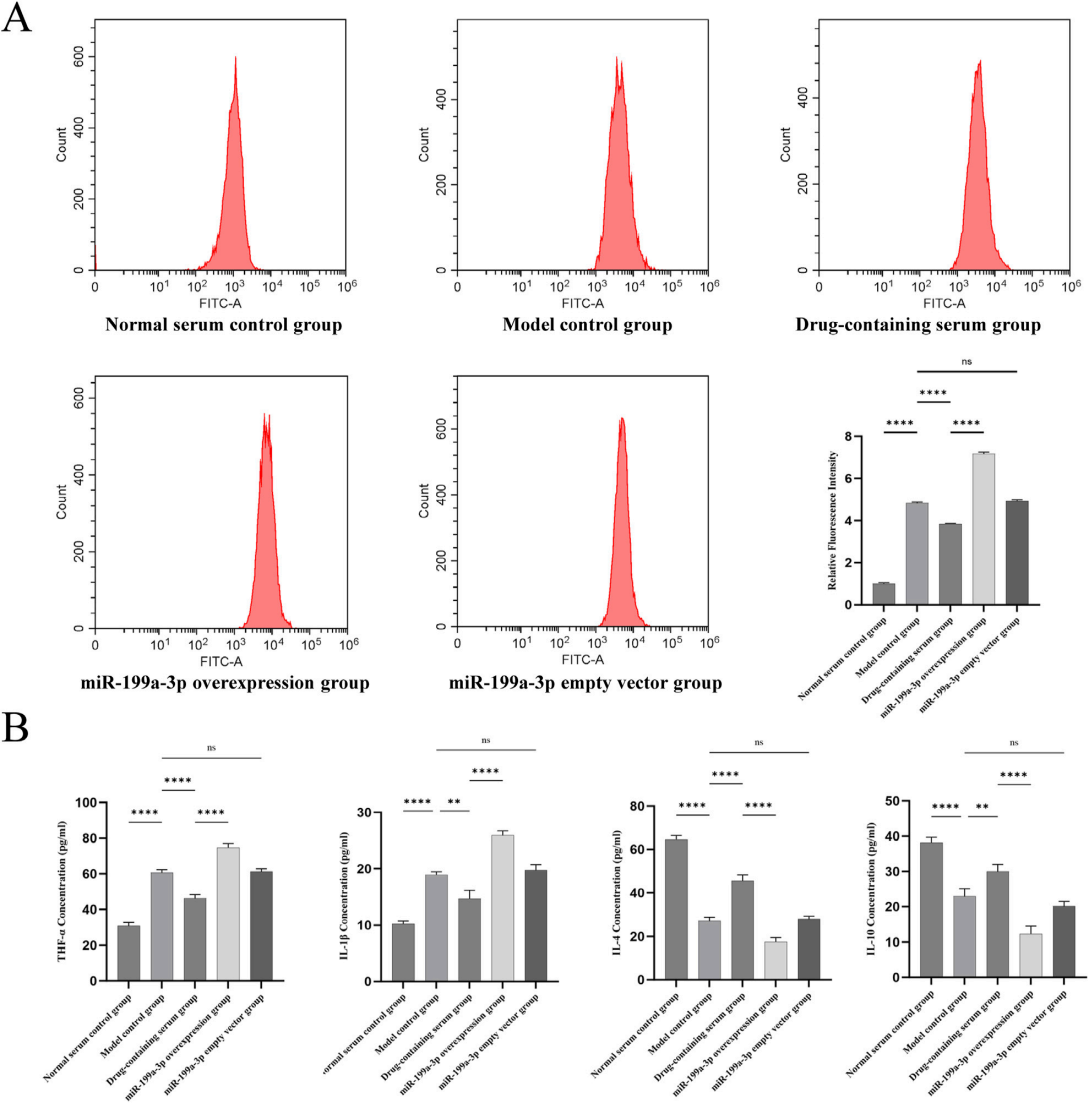
**(A)** Flow cytometric analysis of reactive oxygen species (ROS) levels; **(B)** Results from the ELISA assay quantifying the expression of inflammatory cytokines TNF-α, IL-1β, IL-4, and IL-10 in cultured cells. nsP >0.05; *p < 0.05; **p < 0.01; ***p < 0.001; ****p < 0.0001.

Flow cytometry was employed to detect ROS levels, reflecting the oxidative stress status in the cellular microenvironment. The Model control group exhibited a significant increase in ROS production compared to the Normal serum control group, indicating that T-BHP-induced oxidative stress markedly elevated ROS generation. ROS levels in the Drug-containing serum group were significantly lower than those in the Model control group, suggesting that BSHXF partially mitigated T-BHP-induced oxidative stress. Conversely, the miR-199a-3p overexpression group displayed a further pronounced elevation in ROS content, demonstrating that miR-199a-3p overexpression exacerbated oxidative stress injury and disrupted cellular redox homeostasis (Figure 6A).

ELISA results demonstrated that the expression of pro-inflammatory cytokines TNF-α and IL-1β was significantly upregulated in the Model control group compared to the Normal serum control group, whereas the anti-inflammatory cytokines IL-4 and IL-10 were markedly downregulated. These findings indicate that T-BHP-induced oxidative stress triggered a pronounced inflammatory response, suppressed anti-inflammatory factor expression, and impaired inflammatory regulation. In contrast, the Drug-containing serum group exhibited reduced expression of TNF-α and IL-1β alongside elevated levels of IL-4 and IL-10, suggesting that BSHXF diminished pro-inflammatory cytokine concentrations and partially restored anti-inflammatory factor secretion. The miR-199a-3p overexpression group displayed a further increase in TNF-α and IL-1β expression coupled with a significant decrease in IL-4 and IL-10 levels, demonstrating that miR-199a-3p overexpression exacerbated inflammatory cytokine release and suppressed anti-inflammatory capacity (Figure 6B).

### 3.7 Histopathological evaluation of intervertebral discs

**FIGURE 7:**
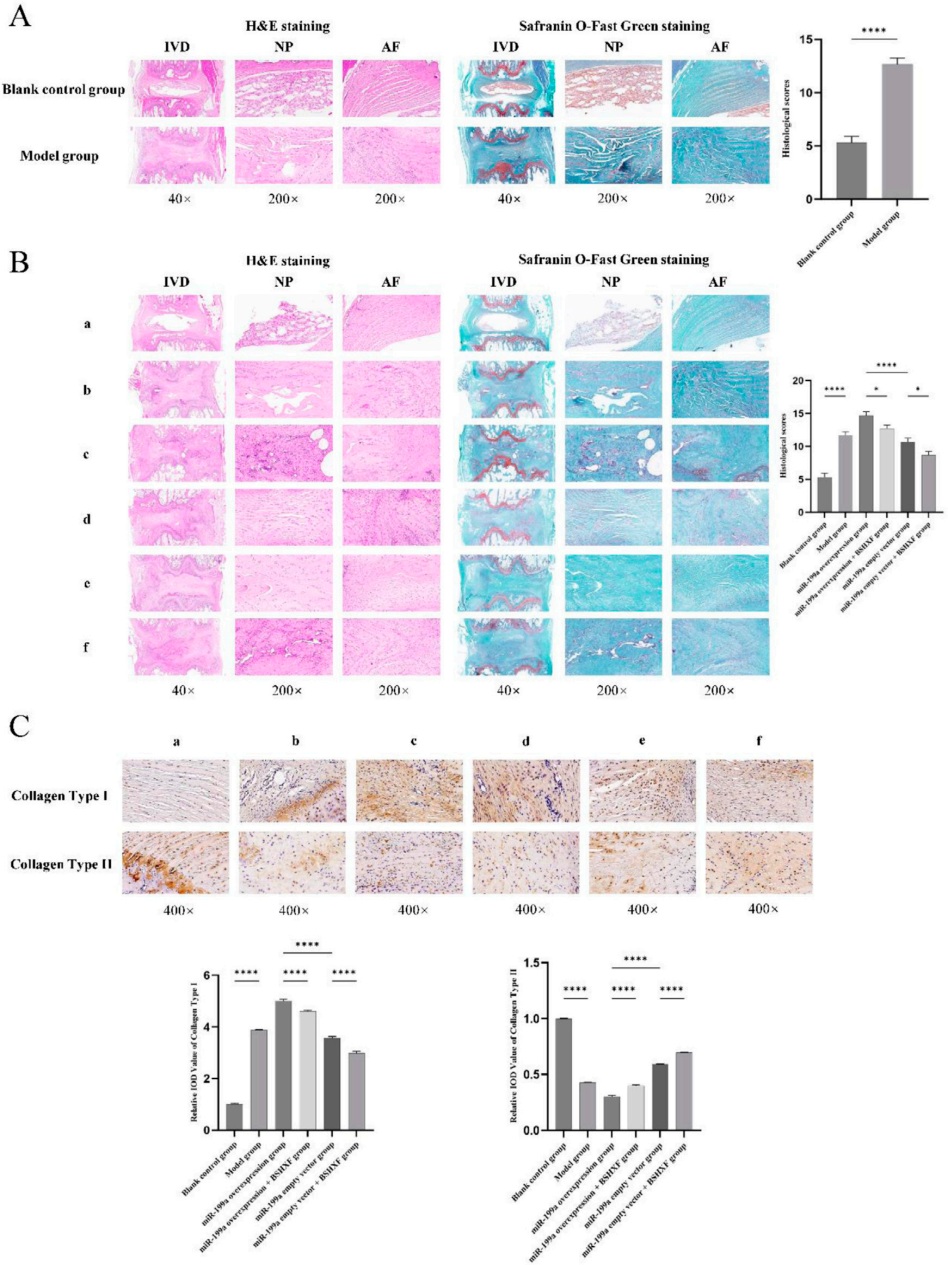
**(A)** Validation of the model using HE staining and Safranin O-Fast Green staining; **(B)** Evaluation of intervertebral disc degeneration in each experimental group through HE staining and Safranin O-Fast Green staining; **(C)** Immunohistochemical analysis of intervertebral discs in each group. **(A)** Blank control group; **(B)** Model group; c:miR-199a-3p overexpression group; d:miR-199a overexpression + BSHXF group; e:miR-199a-3p empty vector group; f:miR-199a empty vector + BSHXF group; nsP >0.05; *p < 0.05; **p < 0.01; ***p < 0.001; ****p < 0.0001.

To validate the successful establishment of the IDD model in rats, histological analysis of caudal intervertebral disc tissues from the Blank control group and Model group was performed at 2 weeks post-modeling using HE and Safranin O-Fast Green staining. This confirmed the feasibility of the model and provided a reliable foundation for subsequent experiments. Both HE and Safranin O-Fast Green staining revealed (Figure 7A) that compared to the Blank control group, the Model group exhibited decreased disc height, structural disruption of the AF and NP, visible AF fissures and serpentine fibers, significant NP cell loss, disrupted AF-NP boundaries, NP invasion by AF tissue, elevated histopathological scores, and marked degeneration, confirming the feasibility of the caudal disc puncture-induced IDD model. After 4 weeks of gavage administration, intervertebral disc degeneration was assessed in all groups via histological staining. The results (Figure 7B) showed that histopathological scores in the Model group were significantly higher than those in the Blank control group, consistent with the 2-week model validation findings. Compared to the miR-199a-3p overexpression + BSHXF group, the miR-199a-3p overexpression group displayed exacerbated disc degeneration, characterized by further reduced disc height, aggravated AF damage, pronounced NP cell depletion, and the highest histopathological scores. BSHXF administration partially alleviated miR-199a-3p overexpression-induced disc degeneration. Furthermore, comparison between the miR-199a-3p empty vector group and miR-199a-3p empty vector + BSHXF group further validated the therapeutic efficacy of BSHXF in mitigating IDD progression. Additionally, IHC assays were conducted on intervertebral disc tissues to evaluate changes in the AF and NP during degeneration by measuring COL1A1 and COL2A1 expression. The results (Figure 7C) revealed that COL1A1 expression was significantly upregulated, while COL2A1 expression was downregulated in the Model group compared to the Blank control group, indicating that puncture-induced modeling increased disc fibrosis and impaired NP functionality. Compared to the miR-199a-3p overexpression + BSHXF group, BSHXF administration via gavage significantly reduced COL1A1 expression and increased COL2A1 levels in the miR-199a-3p overexpression group, suggesting that BSHXF partially alleviated miR-199a-3p overexpression-induced disc fibrosis and NP functional impairment. Furthermore, comparison between the miR-199a-3p empty vector group and miR-199a-3p empty vector + BSHXF group confirmed that BSHXF administration significantly improved collagen expression and attenuated disc degeneration.

### 3.8 Regulatory role of miR-199a-3p in the TGF-β/Smad signaling axis

**FIGURE 8:**
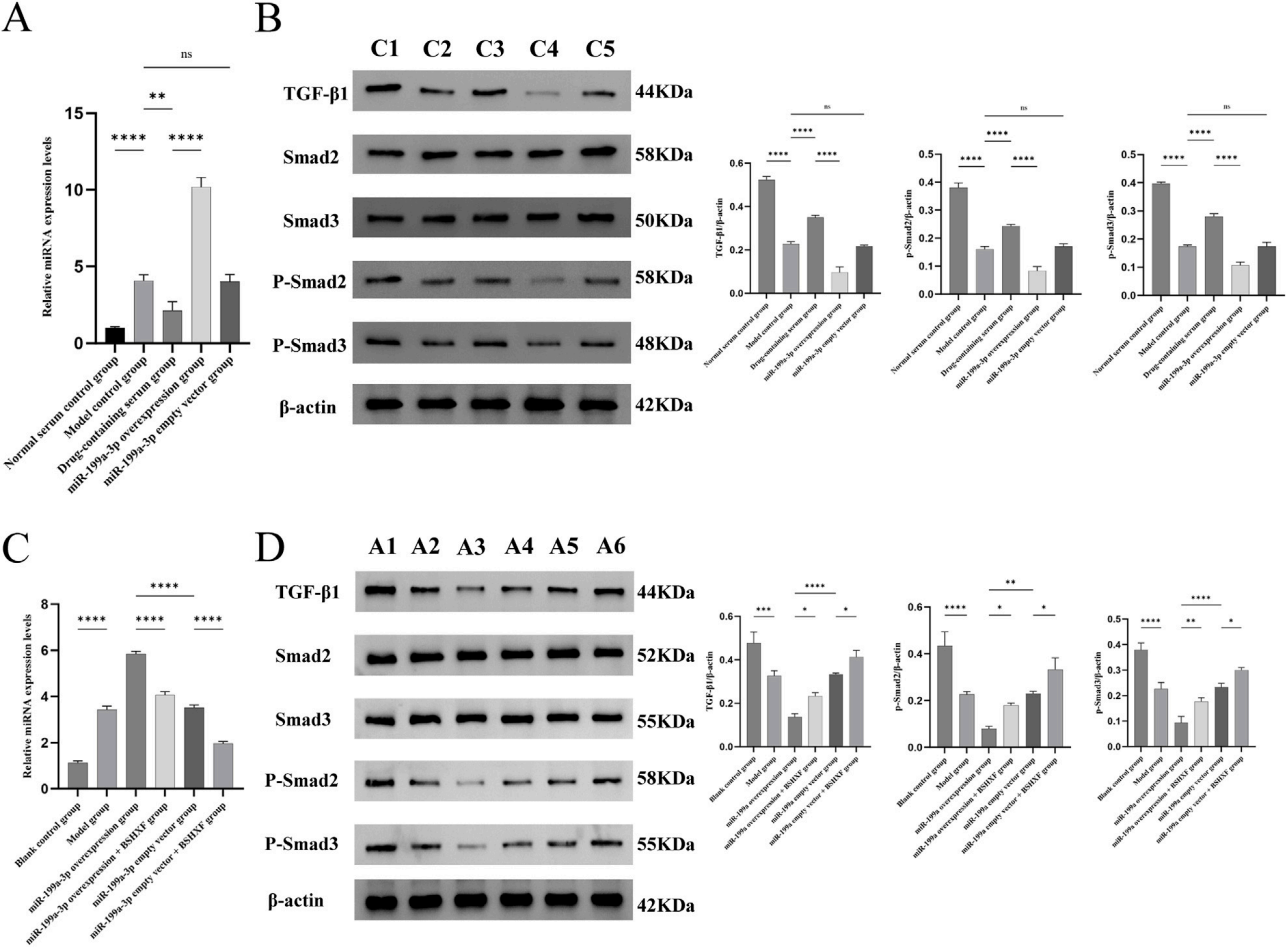
**(A)** Expression analysis of miR-199a-3p in each experimental group from the *in vitro* cell assay; **(B)** Western blot analysis of relevant protein expression in the signaling pathways in the cell experiment; **(C)** Expression analysis of miR-199a-3p in each experimental group from the *in vivo* animal model; **(D)** Western blot analysis of relevant protein expression in the signaling pathways in the animal experiment. C1:Normal serum control group; C2:Model control group; C3:Drug-containing serum group; C4:miR-199a-3p overexpression group C5:miR-199a-3p empty vector group; A1:Blank control group; A2:Model group; A3:miR-199a-3p overexpression group; A4:miR-199a overexpression + BSHXF group; A5:miR-199a-3p empty vector group; A6:miR-199a empty vector + BSHXF group. nsP >0.05; *p < 0.05; **p < 0.01; ***p < 0.001; ****p < 0.0001.

In this study, we evaluated the regulatory role of miR-199a-3p within the TGF-β/Smad signaling axis. In cellular experiments, RT-qPCR results (Figure 8A) demonstrated that miR-199a-3p expression was significantly upregulated in the Model control group compared to the Normal serum control group, whereas it was downregulated in the Drug-containing serum group, reflecting the potential inhibitory effect of BSHXF. The miR-199a-3p overexpression group exhibited the highest miR-199a-3p expression among all groups, confirming successful overexpression. Western blot analysis (Figure 8B) revealed that the expression levels of TGF-β1, P-Smad2, and P-Smad3 were significantly reduced in the Model control group compared to the Normal serum control group, indicating that T-BHP-induced oxidative stress markedly suppressed the activity of these signaling molecules. Following BSHXF treatment, the Drug-containing serum group displayed elevated expression levels of TGF-β1, P-Smad2, and P-Smad3 compared to the Model control group, demonstrating that BSHXF partially reversed T-BHP-induced suppression of this signaling pathway. However, the miR-199a-3p overexpression group exhibited further reductions in TGF-β1, P-Smad2, and P-Smad3 expression, highlighting the negative regulatory role of miR-199a-3p in this critical signaling pathway.

In the animal experiments, we assessed miR-199a-3p expression in the animal model using RT-qPCR (Figure 8C). The Model control group exhibited significantly elevated miR-199a-3p expression compared to the Blank control group, demonstrating that disease model establishment activated miR-199a-3p expression. The miR-199a-3p overexpression group showed significantly higher miR-199a-3p levels than all other groups, confirming the efficacy of the overexpression construct. Following intragastric administration of BSHXF, miR-199a-3p expression in the miR-199a-3p overexpression + BSHXF group decreased, suggesting BSHXF has the potential to modulate miR-199a-3p expression to some extent. Western blot results (Figure 8D) revealed that TGF-β1, P-Smad2, and P-Smad3 expression levels in the Model control group were significantly reduced compared to the Blank control group, indicating that disease model establishment suppressed activation of the TGF-β/Smad signaling pathway. Compared to the miR-199a-3p overexpression group, the miR-199a-3p overexpression + BSHXF group displayed increased TGF-β1, P-Smad2, and P-Smad3 expression after BSHXF administration, demonstrating that BSHFX alleviated the inhibitory effects of miR-199a-3p overexpression. Comparative analysis between the miR-199a-3p overexpression group and the miR-199a-3p empty vector group revealed the direct regulatory impact of miR-199a-3p overexpression on cellular signaling pathways.

## 4 Discussion

This study is the first to elucidate the synergistic mechanism by which BSHXF drug-containing serum combined with ADSCs delays IDD through regulation of the miR-199a-3p/TGF-β/Smad signaling pathway. Characteristic pathological features of IDD include NPCs dysfunction, ECM metabolic imbalance, and a vicious cycle of chronic inflammation and oxidative stress within the microenvironment (Kamali et al., 2021). Although ADSCs possess significant potential to differentiate into various cell types (Mazini et al., 2019), including NPC-specific phenotypes (Huang et al., 2023; Xu et al., 2020), the primary challenge in cell-based therapy lies in ensuring transplanted ADSCs survive, proliferate, and differentiate within the hostile degenerative disc microenvironment (Ma et al., 2019). Recent studies indicate that certain kidney-tonifying and blood-activating herbal compounds enhance the survival and efficacy of transplanted stem cells (Hu et al., 2022; Wang C. et al., 2022), suggesting that traditional Chinese medicine targeting kidney and blood functions may ameliorate degenerative microenvironments and improve ADSCs survival post-transplantation. Integrated cellular and animal experimental data in this study confirm two pivotal findings:1. BSHXF promotes ADSC differentiation into nucleus pulposus-like cells by activating the TGF-β/Smad pathway, while counteracting oxidative stress-induced cell cycle arrest, membrane integrity damage, and senescence.2. miR-199a-3p exacerbates ECM degradation and inflammatory responses via targeted suppression of the TGF-β/Smad pathway, whereas BSHXF restores pathway activity by downregulating miR-199a-3p, thereby significantly improving disc microstructure and inhibiting fibrosis. These findings underscore the critical role of miR-199a-3p in IDD pathogenesis, mediating ECM metabolism and cellular functions through modulation of the TGF-β/Smad pathway.

The deterioration of the intervertebral disc microenvironment restricts the proliferation of endogenous and transplanted cells, as well as their capacity to synthesize ECM. Local oxidative stress and inflammatory responses are critical contributors to this microenvironmental degeneration (Chen H. W. et al., 2023). Oxidative stress induces senescence or apoptosis of endogenous NPCs and suppresses proliferation/differentiation of transplanted cells. Although the intervertebral disc resides in a relatively hypoxic environment, ROS are still generated via oxidative phosphorylation. Under physiological conditions, intracellular ROS production and clearance maintain dynamic equilibrium; disruption of this balance triggers oxidative cellular damage (Sies, 2020). Degenerated discs exhibit markedly diminished antioxidant capacity. For instance, reduced FoxO expression in both human and murine degenerative disc tissues directly suppresses downstream antioxidant proteins (e.g., SESN and SOD2), exacerbating oxidative damage (Alvarez-Garcia et al., 2017). Excessive ROS-mediated oxidative stress elevates disc cell apoptosis and senescence, reduces autophagy activity, and induces structural ECM degradation. These changes diminish disc elasticity, increase stiffness, impair biomechanical function, and ultimately drive IDD (Cheng et al., 2022). Researchers have experimentally demonstrated that oxidative stress elevates intracellular ROS levels in nucleus pulposus-derived mesenchymal stem cells (NP-MSCs), suppresses cell viability, and promotes apoptosis (Chen et al., 2020). In this study, we established a T-BHP-induced oxidative damage model in ADSCs. T-BHP metabolically generates alkoxy radicals and hydrogen peroxide, elevating intracellular ROS levels and causing DNA damage, lipid peroxidation (e.g., increased malondialdehyde), and protein dysfunction (Juan et al., 2021). This oxidative cascade activates DNA damage response pathways (e.g., ATM/Chk2), induces G1 phase arrest by inhibiting CDK4/6-Cyclin D complex activity, and prevents S-phase entry (Ciccia and Elledge, 2010). This study revealed that BSHXF significantly mitigates T-BHP-induced oxidative damage in ADSCs, restores cell viability, alleviates cell cycle arrest and senescence phenotypes, reduces ROS accumulation, and decreases LDH leakage, collectively achieving oxidative repair.

Studies have demonstrated that inflammatory responses drive the initiation and progression of IDD. Pro-inflammatory cytokines such as IL-1β and TNF-α are pivotal contributors to IDD and LBP, closely associated with diverse pathological processes in IDD (Cui et al., 2020). Under mechanical injury, stress, or infection, the AF and NP secrete TNF-α and IL-1β. These cytokines are highly expressed in degenerative IDD tissues and participate in multiple pathological cascades, including ECM degradation, inflammatory amplification, apoptosis, dysregulated autophagy, and aberrant cell proliferation (Wang Y. et al., 2020). Excessive pro-inflammatory cytokines and ECM degradation further accelerate NPCs senescence, thereby exacerbating IDD progression. Furthermore, localized disc inflammation impairs mesenchymal stem cell proliferation and differentiation. TNF-α induces mesenchymal stem cell apoptosis and suppresses their differentiation into NPCs, though activation of the NF-κB inflammatory signaling pathway can mitigate these detrimental effects (Cheng et al., 2019). During IDD progression, oxidative stress and inflammatory responses coexist and synergistically exacerbate disc microenvironmental deterioration. In this study, elevated ROS and inflammatory mediator expression confirmed that T-BHP-induced oxidative stress concurrently activates inflammation. This was evidenced by upregulated ROS levels and pro-inflammatory cytokines (TNF-α, IL-1β), alongside downregulated anti-inflammatory factors (IL-4, IL-10). These findings collectively indicate that oxidative stress and inflammatory responses within the intervertebral disc synergistically disrupt the local microenvironment. Such alterations not only impair endogenous NPCs-mediated repair of degenerative discs but also hinder the proliferation and differentiation of exogenous transplanted cells, such as MSCs and ADSCs, into functional NPCs.

Studies have revealed that among the top signaling pathways potentially regulated by miRNAs, the TGF-β, platelet-derived growth factor (PDGF), insulin-like growth factor (IGF), and epidermal growth factor (EGF) pathways have been implicated in the pathogenesis of IDD (Hegewald et al., 2014; Koerner et al., 2014; Li et al., 2008). The TGF-β/Smad signaling pathway is a central regulator of cell differentiation, proliferation, and ECM synthesis (Du X et al., 2023). TGF-β1, a ubiquitously expressed growth factor, modulates proliferation, migration, differentiation, and survival across diverse cell types (Janssens et al., 2005). Notably, the TGF-β signaling pathway is essential for disc homeostasis. It protects disc tissue by enhancing matrix formation, limiting matrix catabolism, and attenuating inflammatory responses (Chen et al., 2019). Specific inhibition of TGF-β1 signaling significantly suppresses Smad2/3 phosphorylation and nuclear translocation of matrix proteins in nucleus pulposus cells (Yang et al., 2016). Activation of the TGF-β/Smad3 axis increases ACAN synthesis in NPCs, preserving disc hydration, structural integrity, and ECM homeostasis (Wu et al., 2012). These findings collectively highlight the therapeutic potential of TGF-β1 in mitigating IDD progression. Within the disc, the ECM provides structural and biochemical support to cells, with its dynamic equilibrium under physiological conditions ensuring cellular stability. COL2A1 and ACAN are primary ECM components in disc cells, jointly maintaining physiological structure and biomechanical function (Kritschil et al., 2024). COL2A1 counteracts catabolic processes and promotes anabolic pathways to suppress NP degeneration (Jiang et al., 2024), while ACAN similarly supports anabolic metabolism. Together, they preserve tissue architecture and mechanical functionality (Empere et al., 2023). SOX9, a key chondrogenic transcription factor, regulates intracellular ACAN and COL2A1 levels (Tsingas et al., 2020). COL2A1, ACAN, and SOX9 collectively stabilize the disc by promoting ECM synthesis and inhibiting degradation, ensuring metabolic equilibrium. The miRNA investigated in this study, miR-199a-3p, is implicated in modulating apoptosis and inflammatory disorders. Studies demonstrate that miR-199a-3p overexpression suppresses cell proliferation and migration, induces apoptosis, and thereby inhibits non-small cell lung cancer progression (Liu et al., 2022). Additionally, miR-199a-3p overexpression attenuates NF-κB-mediated inflammatory responses (Gonzalez-Lopez et al., 2023). In this study, we found that BSHXF significantly upregulated TGF-β1 and p-Smad2/3 expression, activated the TGF-β/Smad signaling pathway, and promoted ADSCs differentiation into nucleus pulposus-like cells (evidenced by increased CD73/COL2A1 dual-positive cell ratios). Additionally, BSHXF counteracted oxidative stress-induced cell cycle arrest, membrane integrity damage, and cellular senescence. Conversely, miR-199a-3p overexpression markedly suppressed TGF-β1 and p-Smad2/3 protein levels, leading to reduced expression of key ECM synthesis molecules (COL2A1, SOX9, and ACAN) and increased release of pro-inflammatory cytokines (TNF-α and IL-1β). By targeting and inhibiting the TGF-β/Smad pathway, miR-199a-3p exacerbated ECM degradation and inflammatory responses. Notably, BSHXF restored pathway activity by downregulating miR-199a-3p expression.

Multiple collagen types within the intervertebral disc have been studied, with COL1A1 and COL2A1 identified as its primary components (Trefilova et al., 2021). The outer annulus fibrosus extracellular matrix is predominantly composed of COL1A1, characterized by tightly packed fibrils that confer high tensile strength and multidirectional mechanical resilience. However, COL1A1 upregulation is frequently associated with fibrosis and tissue degeneration (Varma et al., 2016). In contrast, the inner AF and NP regions exhibit higher COL2A1 content, which endows the disc with elasticity and shock-absorbing capacity to dissipate compressive forces. Reduced COL2A1 levels are closely linked to NP ECM degradation, dehydration, and IDD (Trefilova et al., 2021). In this study, a needle puncture method was employed to mechanically disrupt AF and NP integrity, recapitulating pathological features of IDD such as ECM degradation, apoptosis, and inflammation. This classical IDD modeling approach offers advantages including technical simplicity, cost-effectiveness, and controlled trauma, reliably inducing disc height loss, tissue fibrosis, and dehydration (Chen Y. et al., 2023; Elmounedi et al., 2023). Our findings demonstrate that miR-199a-3p overexpression suppresses the TGF-β/Smad signaling pathway, leading to AF structural disruption, NP cell depletion, disc height reduction, COL1A1 upregulation, and COL2A1 downregulation. These changes reflect exacerbated disc fibrosis, NP functional impairment, and accelerated degenerative progression. Notably, intragastric administration of BSHXF partially alleviated miR-199a-3p overexpression-induced disc degeneration.

In summary, this study elucidates the potential mechanism by which the combined intervention of BSHXF and ADSCs delays IDD through regulation of the miR-199a-3p/TGF-β/Smad signaling axis. BSHXF exerts multi-target protective effects via the following pathways: (1) activating the TGF-β/Smad pathway to promote ADSCs differentiation into nucleus pulposus-like cells; (2) counteracting T-BHP-induced oxidative stress, thereby alleviating cell cycle arrest and senescence; (3) downregulating miR-199a-3p expression to reverse its inhibition of the TGF-β/Smad pathway, consequently reducing ECM degradation and inflammatory responses. In animal models, this intervention significantly improved intervertebral disc structure and suppressed fibrosis. These findings suggest that the combined application of BSHXF and ADSCs achieves synergistic effects by targeting the miR-199a-3p/TGF-β/Smad axis; however, the precise synergistic mechanisms require further validation through single-factor controlled experiments in future studies. Nevertheless, this study provides a novel theoretical foundation for multi-target intervention strategies against IDD, demonstrating potential for translation into clinical therapeutics.

## Data Availability

The raw data supporting the conclusions of this article will be made available by the authors, without undue reservation.

## Glossary

BSHXF: Bu Shen Huo Xue Fang
IDD: Intervertebral Disc Degeneration
ADSCs: Adipose-derived Stem Cells
ROS: Reactive Oxygen Species
SA-β-Gal: Senescence-Associated Beta-Galactosidase
LDH: Lactate Dehydrogenase
CD73: Cluster of Differentiation 73
COL1A1: Collagen Type I alpha 1
COL2A1: Collagen Type II alpha 1
TNF-α: Tumor Necrosis Factor-alpha
IL-1β: Interleukin-1 beta
IL-4: Interleukin-4
IL-10: Interleukin-10
SOX9: SRY (Sex Determining Region Y)-box 9
ACAN: Aggrecan
TGF-β1: Transforming Growth Factor-beta 1
CCK-8: Cell Counting Kit-8
HE: Hematoxylin and Eosin
IHC: Immunohistochemistry
T-BHP: Tert-Butyl Hydroperoxide
ECM: Extracellular Matrix
LBP: Low Back Pain
NPCs: Nucleus Pulposus Cells
UPLC-Q-TOF-MS: Ultra Performance Liquid Chromatography-Quadrupole Time-of-Flight Mass Spectrometry
AF: Annulus Fibrosus
NPCs: Nucleus Pulposus Cells
NP: Nucleus Pulposus
OD: Optical Density
IOD: Integrated Optical Density
SOD: Superoxide Dismutase
CAT: Catalase
