# Supplementary figures and images for: Investigating the regulation of the miR-199a-3p/TGF-β/Smad signaling pathway by BSHXF drug-containing serum combined with ADSCs for delaying intervertebral disc degeneration
Source: Front Pharmacol. 2025 Apr 28;16:1583635. doi: 10.3389/fphar.2025.1583635 (PMC12067415; doi:10.3389/fphar.2025.1583635)

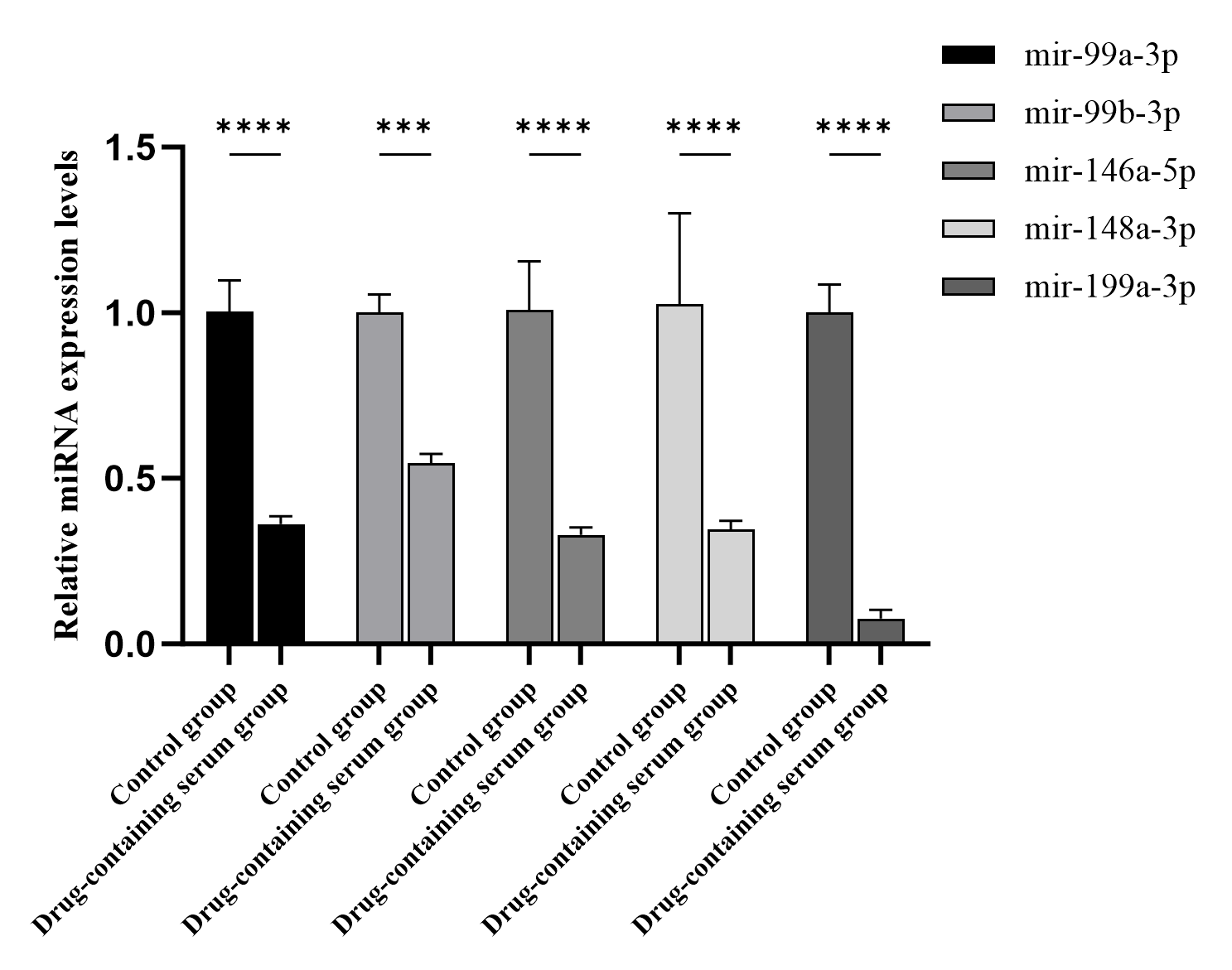

Supplement: Supplementary file 1 [file Image1.tif]
